# Pb Toxicity on Gut Physiology and Microbiota

**DOI:** 10.3389/fphys.2021.574913

**Published:** 2021-03-04

**Authors:** Wenya Liu, Hai Feng, Shuilin Zheng, Shuaishuai Xu, Isaac Yaw Massey, Chengcheng Zhang, Xiaoyan Wang, Fei Yang

**Affiliations:** ^1^Hunan Provincial Key Laboratory of Clinical Epidemiology, Xiangya School of Public Health, Central South University, Changsha, China; ^2^Department of Gastroenterology, Third Xiangya Hospital, Central South University, Changsha, China; ^3^Hunan Province Key Laboratory of Typical Environmental Pollution and Health Hazards, School of Public Health, University of South China, Hengyang, China

**Keywords:** intestinal microbiota, lead, microbial metabolites, gut, toxicity

## Abstract

Lead (Pb) is a toxic heavy metal, having profound threats to the global population. Multiple organs such as kidney, and liver, as well as nervous, hematologic, and reproductive systems, are commonly considered the targets of Pb toxicity. Increasing researches reported that the effects of Pb on gastrointestinal tracts are equally intensive, especially on intestinal microbiota. This review summarized Pb toxicity on gut physiology and microbiota in different animal models and in humans, of which the alterations may further have effects on other organs in host. To be more specific, Pb can impair gut barrier and increase gut permeability, which make inflammatory cytokines, immunologic factors, as well as microbial metabolites such as bile acids (BA) and short-chain fatty acids (SCFAs) enter the enterohepatic circulation easily, and finally induce multiple systematic lesion. In addition, we emphasized that probiotic treatment may be one of the feasible and effective strategies for preventing Pb toxicity.

## Introduction

Lead (Pb), one of the non-essential heavy metals, which is widely considered an environmental pollutant, possesses quite serious health hazards (Watson et al., 2005). In particular, this universal metal exists as a major global public health issue due to its widespread environmental pollution, superior ability to induce a broad spectrum of toxic effects, and the quantity of susceptible demographic groups (Satcher, 2000). It is well known that most urban soils are contaminated with Pb (Mielke et al., 1984; Datko-Williams et al., 2014). Researchers documented that central tendency of Pb concentrations in urban soils ranges from about 100 to 1,000 mg/kg, which is higher than environmental background value (Mielke et al., 1983; Clark et al., 2008; Szolnoki et al., 2013; Mitchell et al., 2014). In recent years, the phasing out of Pb in gasoline and the limitation of Pb content in paint have greatly reduced the heavy metal’s exposure in America (Bellinger, 2016). However, human Pb exposure cannot be simply ignored. Several investigators reported that the average Pb concentration in blood of children in low- and middle-income countries including China (3.71 μg/dl) (Li T. et al., 2017), India (5.46 μg/dl) (Rashid et al., 2019), and Egypt (6 μg/dl) (Mostafa et al., 2009) were higher than that obtained for children in the United States (0.84 μg/dl) (Tsoi et al., 2016). Moreover, Pb exposure can cause health effects at a quite low level (2 μg/dl) (Gilbert and Weiss, 2006). Thus, further reduction is urgently required.

Heavy social burdens of Pb exposure are produced owing to its role in many disorders. Particularly, this heavy metal causes notable neurotoxicity and cognitive developmental problems, for which children are the most susceptible ones than adults (Flora et al., 2012; Evens et al., 2015; Al Osman et al., 2019). Aside from the nervous system, complications of the liver, kidney, hematopoietic, circulatory, cardiovascular, reproductive, gastrointestinal, immunological, and renal systems may also occur from Pb intoxication (Ahamed and Siddiqui, 2007; Navas-Acien et al., 2007; Flora et al., 2012; Riaz et al., 2016). Scholars estimated that Pb exposure significantly increases mortality by 18.0% in the United States (Lanphear et al., 2018).

The mechanisms of Pb’s effects on target organs are universally considered to be general metal toxicity like any other heavy metals. Numerous studies reported that Pb has the capability of relieving oxidative stress (Jomova and Valko, 2011; Huang et al., 2019), inflammation (Metryka et al., 2018), immune response (Gao et al., 2007), and essential metal dyshomeostasis (Liu et al., 2014). In a recent study, the metal was found to function as an endocrine disruptor (He et al., 2017). Unfortunately, these may not address the mechanisms of Pb toxicity; nonetheless, further investigations which will be in favor of appraisal and prevention of the metal’s effects are highly required.

Gut microbiome, well established as our “second genome” (Ley et al., 2006; Zmora et al., 2016), has received increasing attention due to its significant role in a series of physiological functions. For example, regulating host metabolism, immunity, and inflammation, are reactions tightly associated with health and disease (Tremaroli and Backhed, 2012; Dabke et al., 2019). Dysbiosis or disruption of gut microbiota may mediate the process and progress diverse diseases, including allergies (Ver Heul et al., 2019), cancer (Marchesi et al., 2016; Gao R. et al., 2017; Yu and Schwabe, 2017), cardiovascular diseases (Org et al., 2015), obesity (Cavalcante-Silva et al., 2015), intestinal diseases (Ni et al., 2017; Nishida et al., 2018), neurological diseases (Cryan and Dinan, 2012; Mangiola et al., 2016), and diabetes (Wu et al., 2017). Recent reports have suggested gut microbiome as a target for multiple environmental pollutants including persistent organic pollutants, pesticides, antibiotics, nanoparticles, air pollution, endocrine-disrupting chemicals, microplastics, mycotoxins, as well as heavy metals (Becattini et al., 2016; Jin et al., 2017; Velmurugan et al., 2017; Liew and Mohd-Redzwan, 2018; Fackelmann and Sommer, 2019; Yuan et al., 2019; Feng et al., 2020). However, there exists limited knowledge about the possible role of disordered intestinal microbes in Pb toxicity.

Therefore, in this review, we primarily reviewed the effects of Pb exposure on gut microbiota, intestinal structure and function, as well as relevant host health. In addition, we estimated the data for whether gut and its microbiome may be considered mediators for Pb toxicity on other organs, and probiotics treatment may be an operative strategy to prevent Pb toxicity.

## Effect of Pb on Intestinal Microbiota

Gut microbiota has been recognized as an “organ” on itself, which consists of more than 10^14^ microbes and surprisingly possesses 150 times more genes than the human genome (Ley et al., 2006; O’Toole and Jeffery, 2015). Gut microbiome of humans is mainly composed of two major phyla, *Bacteroidetes*, and *Firmicutes*. Some researchers considered the F/B ratio (*Firmicutes* vs. *Bacteroidetes*) crucial to human health, while some do not (Turnbaugh et al., 2009; Zhang et al., 2016; Stojanov et al., 2020). Accumulating literature shows that intestinal microbiota plays a critical part in the development of various diseases, such as cardiovascular diseases, diabetes, obesity, neurological diseases, cancer, and gastrointestinal diseases (Clemente et al., 2012; Lo Presti et al., 2019). Interestingly, gut microbiota also acts as a protective element for heavy metal toxicity, particularly Pb. Increasing reports demonstrated that metabolites derived from microorganisms, including SCFAs, BA, amino acid derivatives, liposaccharides (LPS), and vitamins were crucial signaling molecules linking gut microbiota-host responses (Adak and Khan, 2019; Wang R.X. et al., 2020). The intestinal microbiota is susceptible to the impacts of Pb exposure, generally including alteration of composition, diversity, as well as related metabolites of microbiota.

### Alteration of Gut Microbiota by Pb

Short-term Pb exposure was reported to induce immediate impacts on gut microbiome. In an early study (Sadykov et al., 2009), a significant increase in lactose-negative *Escherichia coli* in the gut microbiota was observed in adult rats after 2 weeks of Pb oral exposure. It is worth knowing that, Pb treatment has profound effects on gut microbiome at phylum, order, and genus levels. It has been demonstrated that Pb (30 μg/L) exposure for 7 days induced significant changes in microbial richness and diversity, a marked increase in *Firmicutes* and *Bacteroidetes*, as well as a significant reduction in *Fusobacteria*, and *Proteobacteria* in gut microbiota on phylum level of zebrafish. Further metabolomics analysis of the liver indicated that a total of 41 metabolites involved in glucose, lipid, amino acid, and nucleotide metabolism were altered. Consistently, the expression of glycolysis and lipid metabolism-related genes, including *Gk*, *Aco*, *Acc1*, *Fas*, *Apo*, and *Dgat*, were notably declined (Xia et al., 2018b). Patsiou et al. (2020) also reported a notable increase in the phylum of *Alphaproteobacteria* and a reduction in *Gammaproteobacteria* of zebrafish following Pb (500 mg/kg) exposure *via* drinking water for 14 days. At the order level, the relative abundance of *Alteromonadales* was decreased with significance. At the genus level, gut microbiota was featured by an upregulation in the relative abundance of *Pseudomonas*, *Halomonadaceae*, *Arcobacter*, and *Polaribacter* (Patsiou et al., 2020). Interestingly, specific *Pseudomonas* strains were reported to be Pb-tolerant bacteria in Pb-contaminated water and soil (Li D. et al., 2017), which revealed that some Pb-against bacteria may exist in zebrafish.

In addition to the short-term Pb exposure, chronic situations are more common. Chronic Pb exposure has been reported to alter both microbial biodiversity and richness to cause dysbiosis of gut microbiota. Cultivable anaerobes increased while cultivable aerobes decreased in the feces of adult mice, which suffered from a constant 2 ppm Pb exposure by drinking water during gestation and lactation periods. In particular, the relative abundance of *Firmicutes* significantly increased, while *Bacteroidetes* exhibited the opposite trend, which indicated that the Pb exposure enhanced the abundant ratio of *Firmicutes* vs. *Bacteroidetes* (F/B) to a certain extent (Wu et al., 2016). In some views, an abnormal abundant ratio of F/B is recognized as a vital biomarker of adiposity and lipid metabolic disorder (Turnbaugh et al., 2009; Stojanov et al., 2020). Moreover, the authors also detected a total of six taxonomic genera change by early life Pb exposure, with three genera dropped (*Lactococcus*, *Enterorhabdus*, and *Caulobacterales*) and three raised (*Desulfovibrionaceae*, *Barnesiella*, and *Clostridium* XIVb). *Desulfovibrio* was reported to convert choline to trimethylamine (TMA), which further oxidized to TMA N-oxide (TMAO) in liver. Accumulation of TMAO was highly correlated to colon cancer and cardiovascular diseases (Baker et al., 1962; Tang et al., 2013; Bae et al., 2014). *Lactococcus* was reported to be probiotics being widely applied in food (Juturu and Wu, 2018). Further correlation analysis revealed that these alterations of microbiota were highly correlated to increased body weight in only males. Oppositely, the F/B ratio showed an evident reduction in the gut microbiota of Japanese quails after 49 days of Pb (1,000 ppm) (Kou et al., 2019). In particular, at the genus level, intestinal bacterial communities were characterized by a remarkable increase in the relative abundance of *Bacteroides*, and a reduction of *Faecalibacterium* and *Bifidobacteria*, accompanied with disrupted intestinal structure and altered immune status. There also exist studies that reported controversial data for the F/B ratio (Breton et al., 2013b; Xia et al., 2018a; Kou et al., 2019). Thus, whether the F/B ratio is a fair index for Pb’s toxic effects needs further explorations. Breton et al. (2013b) investigated the fecal and cecal microorganisms of Balb/C mice affected by Pb at an environmental dose (100 and 500 ppm) *via* drinking water for 8 consecutive weeks. Minor but specific alterations in gut microbiota at both family and genera levels were observed based on 16S rRNA pyrosequencing. The abundance of *Lachnospiraceae* was reduced, whereas the abundance of *Lactobacillaceae* and *Erysipelotrichaceae* were enhanced in the feces and cecum contents of Pb-administrated mice as compared with the control group. Zhai et al.’s (2019c) study also revealed a significant increase of *Ruminococcus* and a decrease of *Turicibacter* in the intestinal microbiome on genera level following C57BL/6 mice exposure to Pb (1 g/L) in drinking water for 8 weeks. However, findings of the changes in *Bacteroidetes* and *Firmicutes* were of no significance. In the same year, Cheng et al. (2019) also observed that Pb resulted in notable changes in gut microbiome of Kunming mice following the same Pb exposure duration and dose, which were distinguished by a significantly higher population of *Lachnospiraceae_NK4A136_group*, and a lower population of *Helicobacter*, together with structure and function damage of kidney and liver, as well as cognitive impairment. There were no consistent changes of microbes in these two studies, which might be attributed to the distinct species of mice. Zhai’s study demonstrated that alteration in the intestinal microbiome induced by Pb exposure was much more obvious in the first 4 weeks as compared with the latter 4 weeks (Zhai et al., 2017a). The authors further suggested the existence of a sensitive period of gut microbiota to Pb. Another interesting phenomenon was that Pb exposure dominantly reduced the population of certain bacteria. For example, eight core OTUs were significantly reduced at the end of 8 weeks’ Pb exposure, whereas no significant upregulation was observed in any genera. Consistent with Monast’s report, chronic Pb exposure reduced the relative abundance of *Ruminococcaceae* family strains, and resulted in further gut dysfunction (Monast et al., 2016), and chronic Pb exposure reduced the relative abundance of *Ruminococcaceae* family strains, which may result in further gut dysfunction. Interestingly, the abundance of *Akkermansia*, considered a marker for colitis (Wang L. et al., 2020), was pronounceably downregulated. According to previous reports, the alterations of these core gut strains were correlated with intestinal inflammation, colitis, and other gut disorders (McLellan et al., 2013; Schwab et al., 2014; Breton et al., 2016). For example, *Oscillibacter* strains were reported to be valeric acid producing bacteria, possessing the potential to be negatively related to gut dysfunction (Park et al., 2014). Certain *Lachnoclostridium* strains were involved in the production of secondary bile acids (Ridlon et al., 2015). Furthermore, a significant reduction in the abundance of *Ruminoclostridium* in IBD patients has been reported (Monast et al., 2016). In addition, a significant decline in diversity of intestinal microbiome was detected by Giri et al. (2018), especially the population of lactic acid bacteria (LAB) in *Cyprinus carpio* fish, exposed to Pb (1 mg/L) for 6 weeks. Gao B. et al. (2017) adopted the 16S rRNA sequencing as well as metagenomics to examine the gut microbiota of mice stool after Pb (10 ppm) exposure by drinking water for 0, 4, and 13 weeks. As expected, the α-diversity and microbiome community structures (assessed by β-diversity metrics) at weeks 4 and 13 were significantly altered. Moreover, the changes of gut microorganisms were almost consistent with Zhai et al.’s (2017a) study. With regard to chronic exposure to low Pb concentration in mice (0.1 mg/L Pb for 15 weeks), the β-diversity was significantly changed (Xia et al., 2018a). Specifically, the relative abundance of *Firmicutes* was reduced, whereas *Bacteroidetes* was increased, leading to a downregulation of F/B ratio. At genus level, the percentage of *Parabacteroides* strains increased, while the percentage of *Dehalobacterium* population dropped. *Parabacteroides*, considered opportunistic pathogens, are generally involved in infectious diseases such as intra-abdominal processes and bacteremia (Aldridge, 1995). Moreover, some critic genes related to hepatic lipid metabolism were notably upregulated, consisted with increased hepatic total cholesterol (TCH) and triglyceride (TG) levels, indicating a high-relevant status between changed gut microbes and hepatic function disturbance (Aldridge, 1995).

Exposure to Pb can also disturb the homeostasis of human intestinal microorganisms. Accumulated reports have demonstrated that Pb exposure has profound negative impacts on human gut microbiota both in adults and children. Bisanz et al. (2014) discovered that increasing blood Pb levels were related to a higher relative abundance of *Succinivibrionaceae* and *Gammaproteobacteria* in feces among Tanzanian pregnant women and school children. Recently, Eggers et al. (2019) investigated urinary Pb concentration and composition of gut microbiota in 696 adult participants in the Survey of the Health of Wisconsin (SHOW) cohort. Results suggested positive associations between α-diversity, richness of gut microbiome, and urinary Pb levels, while different microbial β-diversity were linked to different urinary Pb levels. In particular, the upregulated percent of *Proteobacteria* population, including members of the *Burkholderiales*, were significantly related to increased urine Pb. Shao and Zhu (2020) also adopted 16S rRNA sequencing to investigate the gut microbiota of residents surrounding a mining and smelting area. The microbial diversity and composition profile were altered due to long-term exposure to multiple metals including Pb (Shao and Zhu, 2020). Additionally, a higher abundance of *Lachnospiraceae*, *Eubacterium eligens*, *Ruminococcaceae UGG-014*, *Erysipelotrichaceae UCG-003*, *Tyzzerella 3*, *Bacteroides*, *Slackia*, *italics*, and *Roseburia* and lower abundance of *Prevotella 9* were observed in the stool of various metal exposure participants. Moreover, there existed some differences between females and males, in which microbial richness and evenness were greater for men who received long-term metal exposure in the mining and smelting areas. Reports indicate that, *Lachnospiraceae*, *Erysipelotrichaceae*, and *Eubacterium eligens* are usually considered to be linked to intestinal inflammation. While *Roseburia* is associated with immunity maintenance and anti-inflammatory properties, *Ruminococcaceae*, is majorly associated with mucosa, and is generally recognized as beneficial bacteria (Tamanai-Shacoori et al., 2017).

Therefore, Pb exposure does have varying degrees of impacts on intestinal flora no matter the animal model or human on the basis of substantial studies, and are summarized in Table 1. More generally, a marked increase in the F/B ratio was suggested in most of the previous studies, attributed to increased *Firmicutes* population and/or decreased *Bacteroidetes* population. However, there are some other studies with the opposite results and the rest with no significant changes. In addition, some key bacteria producing SCFAs, BA, and/or other materials related to host disease were differently altered after Pb exposure, such as *Desulfovibrio*, *Lactococcus*, *Akkermansia*, *Ruminococcus*, and *Turicibacter*. The existing contradictions of Pb effects on specific intestinal microbes may be attributed to the distinct exposure dose, time duration, exposure period of life, different animal models, or different subjects.

**TABLE 1:** Effects of Pb on gut microbiome and host.

Model	Method	Dose	Time	Gut microbiota	Outcomes	References
Mice	Drinking water	2 ppm	Gestation and lactation	Cultivable anaerobes↑ Cultivable aerobes↓ **Phylum:** *Firmicutes*↑ *Bacteroidetes*↓ No significant changes in richness and diversity **Genus:** *Desulfovibrionaceae*, *Barnesiella*, *Clostridium XIVb*↑ *Lactococcus*, *Akkermansia Enterorhabdus*, *Caulobacterales*↓	Adult bodyweight change (male offspring)	Wu et al., 2016
Mice	Drinking water	10 ppm	4, 13 weeks	**4 weeks** **Genus:** *S24-7_g*↑ *Clostridiales_f_g*, *Lachnospiraceae_Ruminococcus*, *Ruminococcaceae_g_*, *Ruminococcaceae_Oscillospira*, *Ruminococcaceae_Ruminococcus*↓ **13 weeks** **Genus:** *Clostridiaceae_g_*↑ *Lachnospiraceae_Other*, *Lachnospiraceae_Blautia*, *Lachnospiraceae_Coprococcus*, *Lachnospiraceae_Ruminococcus*, *Ruminococcaceae_Other*, *Ruminococcaceae_Ruminococcus*↓	Numerous microbial metabolic pathways alteration (vitamin E, bile acids, nitrogen metabolism, energy metabolism, oxidative stress, and the defense/detoxification mechanism)	Gao B. et al., 2017
Mice	Drinking water	1 g/L	4 weeks, 8 weeks	**8 weeks Phylum:** *Proteobacteria, Verrucomicrobia*↓ **Genus:** *Parabacteroides*↑; *unclassified and uncultured Ruminococcaceae*, *Lachnospiraceae_incertae_sedis*, *Ruminiclostridium_9*, *Rikenellaceae_RC9_gut_group*, *Oscillibacter*, *Anaerotruncus*, *Lachnoclostridium*, *Akkermansia*↓	/	Zhai et al., 2017a
Mice	Drinking water	0.1 mg/L	15 weeks	**Phylum:** *Bacteroidetes*↑ *Firmicutes*↓ **Genus:** *Parabacteroides*↑ *Dehalobacterium*↓	Hepatic lipid metabolism disruption Microbial metabolism alteration	Xia et al., 2018a
Mice	Drinking water	1 g/L	8 weeks	**Genus:** *Ruminococcus*↑ *Turicibacter*↓	Gut permeability↑ (Muc2, ZO-1, claudin-1, and occludin↓)	Zhai et al., 2019c
Mice	Drinking water	1 g/L	8 weeks	**Phylum:** *Helicobacter*↓ *Lachnospiraceae_NK4A136_group↑*	Hepatotoxicity, nephrotoxicity, oxidative damage; SCFAs (acetate, propionate, and butyrate)↓	Cheng et al., 2019
Japanese quails	Drinking water	50, 1,000 ppm	49 days	**1,000 ppm** **Phylum:** *Bacteroidetes*↑ *Firmicutes*, *Actinobacteria*↓ **Genus:** *Bacteroides*↑ *Faecalibacterium*, *Bifidobacteria*↓	Cecal structure disruption, intestinal inflammation, and immune disorder	Kou et al., 2019
Mice	Drinking water	100 ppm, 500 ppm	8 weeks	**100 ppm** **Family:** *Lactobacillaceae*, *Erysipelotrichaceae*↑ *Lachnospiraceae*↓ **Genus:** *Turicibacter*↑ *Barnesiella*, *Alistipes*↓	/	Breton et al., 2013b
Rats	Oral	333.21, 77.75, 19.44, and 4.86 mg/kg	2 weeks	*Lactose-negative E. coli↑*	*E. coli* attachment↑	Sadykov et al., 2009
Zebrafish	Water	10 and 30 μg/L	7 days	**30 μg/L** **Phylum:** *Firmicutes*, *Bacteroidetes*↑ *Fusobacteria*, *Proteobacteria*↓	Hepatic metabolism disturbance (glucose and lipid metabolism, amino acid metabolism, nucleotide metabolism)	Xia et al., 2018b
Zebrafish	Food	500 mg/kg	14 days	**Phylum:** *Alphaproteobacteria*↑ *Gammaproteobacteria*↓ **Order:** *Alteromonadales*↓ **genus:** *Pseudomonas*, *Halomonadaceae*, *Arcobacter*, *Polaribacter↑*	/	Patsiou et al., 2020
Human	/	/	/	*Succinivibrionaceae*, *Gammaproteobacteria↑*	/	Bisanz et al., 2014
Human	/	/	/	*Proteobacteria*, *Burkholderiales↑*	/	Eggers et al., 2019
Human	/	/	/	*Lachnospiraceae*, *Eubacterium eligens*, *Ruminococcaceae UGG-014*, *Erysipelotrichaceae UCG-003*, *Tyzzerella 3*, *Bacteroides*, *Slackia*, *italics*, and *Roseburia*↑ *Prevotella 9*↓	/	Shao and Zhu, 2020

### Alteration of Metabolism Associated With Gut Microbiota by Pb

Microbial metabolites can interact with the host system locally and systemically, which may trigger intensive biological effects (Holmes et al., 2011). Changes of relative abundance in gut flora consequently influence the microbial metabolic profiles (Sharon et al., 2014; Adak and Khan, 2019). Typical intestinal microbial metabolites such as bile acids and SCFAs, generally act as signaling molecules and bind to cellular receptors (Koh et al., 2016; Wahlström et al., 2016; Wang R.X. et al., 2020). Specifically, bile acids can bind to GPCR TGR5 and nuclear receptor farnesin X receptor (FXR), and SCFAs can bind to G-protein-coupled receptors (GPCRs) (Tremaroli and Backhed, 2012). In addition, upregulation of SCFAs (especially butyric acid) can lower the pH of intestinal lumen and modulate the mucin synthesis and secretion, which may further enhance the intestinal barrier function (Wong et al., 2006; Burger-van Paassen et al., 2009). Activation of signaling pathways may trigger crucial biological effects. Pb exposure is found to have impacts on gut bacteria and some metabolic pathways. Gao B. et al. (2017) adopted multi-omics approaches and elucidated alteration of the microbiome and relevant metabolic profile of C57 BL/6 mice induced by Pb (10 ppm) for 4 and 13 weeks. Data showed that microbial metabolic profiles were seriously perturbed by Pb exposure. In particular, 1,314 molecular features with *p* < 0.05 and fold changes > 1.5 were identified, and specifically considerable metabolic pathways, including vitamin E, bile acids, nitrogen metabolism, energy metabolism, oxidative stress, and the defense/detoxification mechanism, were changed. The levels of vitamin E and bile acids were significantly disturbed. For example, α-tocopherol and γ-tocopherol, the primary bile acids cholic acid and ursodeoxycholic acid, the secondary bile acid deoxycholic acid, and cholesterol, as well as its derivative coprostanol, were notably reduced in Pb-treated group. Nitric oxide (NO) plays a key role in physiological and pathophysiological events in the gut (Cho, 2001; Lamattina et al., 2003). It was further suggested that Pb exposure may increase NO generation in the gut bacteria for gene encoding copper-containing nitrite reductase upregulated after 13-week Pb exposure. Applying nuclear magnetic resonance (^1^NMR) analysis, Xia et al. (2018a) observed that 15 metabolites in cecum contents were significantly changed in mice exposed to a low concentration of Pb (0.1 mg/L) in drinking water for 15 weeks. An increase in the levels of 4-guanidinobutyrate, choline, citrate, glutamate, isobutyrate (a short-chain fatty acid, SCFA), and lysine, as well as a decrease of alanine, glycine, isoleucine, leucine, phenylalanine, tyrosine, valine, and β-galactose were noted. Furthermore, these microbial metabolites were involved in amino acid metabolism, the tricarboxylic acid cycle (TCA cycle), and energy metabolism in host. Interestingly, the related metabolic genes in the host exhibited consistent changes with microbial metabolites. For example, the genes related to lipid metabolism in the liver, some involved in both *de novo* fatty acids synthesis and transport pathways as well as genes involved in TG synthesis were upregulated in a dose-dependent manner in liver of mice treated with Pb. Thus, the authors hypothesized that the gut microbiome and its metabolites might be closely associated with the perturbation of lipid metabolism in mice exposed to Pb. Cheng et al. (2019) also demonstrated that the levels of fecal SCFAs, such as propionate, butyrate, and acetate, were notably reduced in mice after 8-week Pb exposure compared with the control group. These findings are in agreement with Zhai et al.’s (2019b) earlier study.

Taken together, the evidences above support that Pb exposure has the ability to induce dysbiosis of gut flora as well as their metabolites, which may consequently interact with host metabolism, and therefore contribute to host health. However, the specific microbe and its metabolites were not fully elucidated in these researches. It is speculated that some important key microbes, not all the microbiota, affect the intestinal health as well as host health. Multi-omics such as metagenome, metabolome, and transcriptome may need further application to investigate the progress profoundly.

## Effects of Pb on Intestinal Barrier

It has been reported that Pb exposure significantly affected the structure and barrier function of the small intestine in rats. Specifically, rough surface villi, extensive areas with degenerative lesions, and microvilli of enterocytes within these areas sometimes completely absent were observed by scanning electron microscopy (Tomczok et al., 1988). However, the results of Breton’s research (Breton et al., 2013a) held a relatively harmless view of Pb toxicity on gut barrier and permeability. Histological features of the ileum, duodenum, and colon of mice administrated with Pb (100 and 500 mg/L) for 4, 8, and 12 weeks *via* drinking water were consistent with the control group. In particular, the length of villi, depth of crypts, and number of goblet and Paneth cells in the small intestine were unchanged. Moreover, genes related to intestinal barrier functions such as *ZO-1*, *Foxp3*, and *Foxo4* in different intestinal parts were almost unchanged by Pb. Regarding the inflammatory-related gene expression, a significant reduction for both proinflammatory mRNA (*Il1b*, *Tnf*, and *Ifng*) and anti-inflammatory mRNA (*Tgfb* and *Il-10*) were detected. Their later study (Breton et al., 2016) demonstrated that transepithelial electric resistance was reduced in human cell-based models. Zhai et al.’s (2019c) research showed that oral administration of high concentration of Pb (1 g/L) for 8 weeks in mice remarkably upregulated the levels of serum DX-4000-FITC and downregulated the mRNA expression of colonic *Muc2* as well as intestinal tight junctions (TJ), including *ZO-1*, *claudin-1*, and *occludin*, accompanied with an increasing abundance of *Ruminococcus* and a decreasing abundance of *Turicibacter*, indicating severely destroyed gut barrier and permeability. This team further evaluated Pb toxicity on gut permeability in mice at colon and small intestine, respectively. The gene expression level of *ZO-1*, *ZO-2*, *claudin-1*, and *occludin* in both colon and small intestine decreased significantly in Pb-treated mice (Zhai et al., 2019b). In addition, an interesting phenomenon was observed by the authors. The mRNA expression of tight junction was further downregulated in Pb-treated mice with a predepletion of gut microbiome. This result gave an evidence that intestinal commensal bacteria might compete with gut for Pb absorption, thereby limiting the bioavailability of Pb. Kou et al.’s (2019) findings also indicated that chronic Pb exposure had intensive effects on cecum histology of Japanese quails, including mucosa abscission, Lieberkühn glands destruction, and lymphocyte hyperplasia. Ultrastructural damages characterized by nucleus pyknosis, mitochondrial vacuolation, and microvilli contraction were further observed *via* transmission electron microscope. Moreover, changes in cecum morphology was related to downregulated expression levels of *IL-2* and *IFN-*γ, while upregulated levels of *IL-6*, *TNF-*α, and *NF-*κ*B*, accompanied by increased *Bacteroides* whereas decreased *Faecalibacterium* and *Bifidobacteria* abundance (Supplementary Figure 1).

## Gut-Liver Axis

The enterohepatic circulation has been intensively reported in previous studies, by which numerous materials in gut such as bile acids (linkage of gut-liver axis) can play vital roles in lipid metabolism, heavy metal excretion, glucose homeostasis, hepatic bile formation, and intestinal function in the host (Jones et al., 2008; Hofmann and Hagey, 2014; Ridlon and Bajaj, 2015). An investigation implemented by Ma et al. (2018), using liver cancer model showed that primary BA accumulation increased natural killer T cell population and inhibited liver tumor growth. Increasing studies indicate that the intestinal microbiome performs a critical role in regulating bile acid homeostasis (Tremaroli and Backhed, 2012; Adak and Khan, 2019). Pb treatment in mice significantly changed intestinal microbial composition as well as mRNA expression of BA metabolic genes such as *Cyp8b1* in the liver and *Fgf15* in the ileum (Zhai et al., 2019c). Xia et al. (2018a) reported that chronic Pb exposure led to liver metabolism perturbance, including lipid metabolism and TCA circle, accompanied by alteration of gut microbes and key genes related to lipid metabolism. Moreover, the authors further detected marked changes in hepatic metabolism and core genes associated with hepatic lipid and glycolysis metabolism (*Gk*, *Aco*, *Acc1*, *Fas*, *Apo*, and *Dgat*) in Pb-treated zebrafish (Xia et al., 2018b). Moreover, relative abundance of certain gut microbes were changed and were reported to be tightly associated with lipid and glycolysis metabolism, disease and inflammation, such as *Bacteroides*, *Flavobacterium*, *Roseburia*, *Alloprevotella*, and *Ruminococcus*. Zhai et al. (2019b) reported a significant reduction of SCFAs, especially butyric acid, when mice were orally exposed to Pb (1,304 mg/kg) for 3 days. Use of antibiotics in mice model of liver disease suggested that depletion of intestinal flora can alleviate liver inflammation by reducing the transport of lipopolysaccharide in liver (Tripathi et al., 2018). As described in Sections “Effect of Pb on intestinal microbiota” and “Effects of Pb on intestinal barrier,” these evidences indicate that the manipulation of gut microbiota composition, diversity, or richness affects BA, SCFA metabolism, and/or other metabolisms and may mediate the interactions between the gut and liver.

## Probiotics Treatment as a Promising Pb-Against Strategy

Quite a few studies about probiotics treatment functioning as a therapeutic method in alleviating Pb toxicity have been published (Supplementary Figure 2), as Pb is considered one of the most toxic heavy metals. Topcu and Bulat (2010) firstly investigated the Pb-removal properties of *Enterococcus faecium* strains (*E. faecium* EF031 and *E. faecium* M74) in water. The preliminary results suggested that both two *E. faecium* stains could bind the Pb efficiently. Bhakta et al. (2012) isolated and identified some Pb-removal lactic acid bacteria (LAB) from the environment, of which *E. faecium* Pb12 strains were the most excellent stains exhibiting the potential for uptaking Pb from fish intestine and further reducing Pb bioaccumulation in tissues and organs. In a recent study, Giri et al. (2019) reported that *Lactobacillus reuteri* P16, isolated from intestinal contents of *Cyprinus carpio* fish, possessed efficient Pb-binding ability (>15% Pb removal), extraordinary antioxidant activity (hydroxyl radical-scavenging, 42.18%), as well as satisfactory probiotics properties (high tolerance to both bile and acid *in vitro*). Daisley et al. (2019) also adopted a Caco-2 model of the intestinal epithelium and investigated the anti-Pb property of *Lactobacillus rhamnosus* GR-1 (LGR-1). The results showed that Pb formed anomalous cell-surface clusters on LGR-1. LGR-1 possessed the ability to effectively reduce apical-to-basolateral translocation of Pb, which thus availably reduced Pb translocation across the intestinal epithelium. Regarding probiotics against Pb toxicity *in vivo*, multiple investigations have also been conducted in the past few years. Having identified *Lactobacillus reuteri* P16 as an effective Pb-removal strain, Giri et al. (2018) further explored its therapeutic effects for waterborne Pb toxicity in fish (*Cyprinus carpio*). The fish were exposed to waterborne Pb (1 mg/L) with *L. reuteri* P16 (10^8^ CFU/g) supplementary diet provided for 6 weeks. Results demonstrated that this probiotic could decrease mortality and Pb accumulation in tissues, improve the growth performance, as well as partly reverse Pb-induced alterations of host, including biochemical parameters, oxidative stress, intestinal enzymatic activities, intestinal microbiota, and expressions of proinflammatory cytokines (*TNF-*α and *IL-1*β) and heat shock proteins (*HSP70* and *HSP90*). Yi et al. (2017) also isolated a lead-resistant LAB strain 96 from a portion of Korean fermented food, identified as *Leuconostoc mesenteroides*. Use of *Leuconostoc mesenteroides* significantly decreased the levels of glutamate oxaloacetate transaminase and glutamate pyruvate transaminase, and particularly restored partial male reproductive function such as the ATP content and mobility of epididymal spermatozoa in Pb-administrated mice. Correspondingly, oral supplementation of Pb-intolerant gut microbiota (*Oscillibacter ruminantium*, *Faecalibacterium prausnitzii*, and *Akkermansia muciniphila*, especially *O. ruminantium* and *F. prausnitzii*) in Pb-exposed mice markedly enhanced the excretion of Pb and increased the expression of tight junction proteins to alleviate the disruption of gut permeability. Notably, SCFAs were upregulated by colonic microbiota, which allowed the authors to propose that probiotics possess the Pb-against potential by regulating intestinal microbiome (Zhai et al., 2019b). Moreover, Raghuvanshi et al. (2017) developed a transgenic probiotic that markedly prevented LPS-induced impairment in rats exposed to Pb, as well as recovered the normal absorption of essential metal ions. In a previous study, Tian et al. (2012) firstly gave evidence that *Lactobacillus plantarum* CCFM8661 could bind Pb *in vitro*, and which also offered remarkable protective effects on hematology and oxidative stress in mice. In a subsequent study, *L. plantarum* CCFM8661 supplementation effectively reversed Pb-induced innate immune status and reduced the frequency of erythrocyte nuclear abnormalities in peripheral blood of fish (Zhai et al., 2017b). The studies above uniformly indicate that the protective effect of probiotics against Pb toxicity might be related to direct biding of the metal *via* a rapid and metabolism-independent surface process or co-precipitation with Pb in the gut (Beyenal and Lewandowski, 2004; Halttunen et al., 2007; Tian et al., 2012). In Zhai et al.’s (2019a) follow-up study, it was found that enterohepatic circulation was modulated by probiotics (*L. plantarum* CCFM8661), which increased bile flow and biliary glutathione output, finally leading to enhanced biliary Pb output and fecal Pb excretion. Furthermore, the expression of target genes in the enterohepatic farnesoid X receptor–fibroblast growth factor (FXR-FGF15) axis was related to this effect, and the use of FXR agonist reversed the effect significantly. In addition, mixed dietary supplements containing probiotics as well as other substances may function as Pb-against material better, which even make a recovery of learning and memory capacities of mice exposed to Pb (Zhai et al., 2018).

## Conclusion and Future Perspective

**FIGURE 1:**
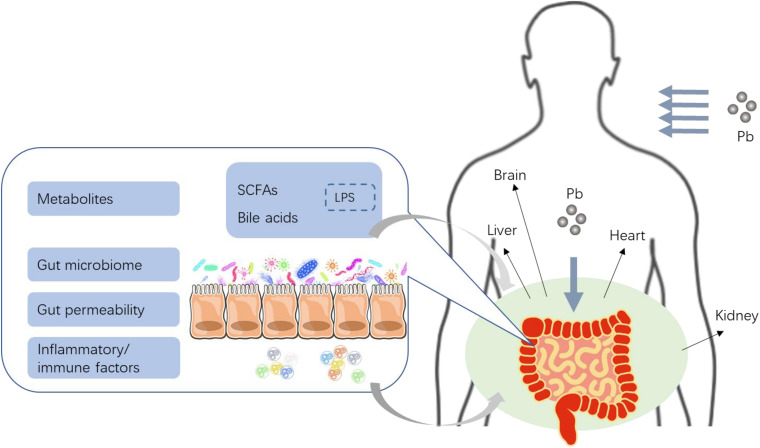
The proposed effects of Pb toxicity on gut, and the critical role of gut barrier and microbiome in Pb toxicity (reproduced from Yuan et al., 2019). Pb entered the gut then changed the microbial homeostasis, including the balance of gut microbe and microbial metabolites such as SCFAs, BA, and/or LPS. Local inflammation and oxidative stress of gut could be induced by Pb generally. Pb could disrupt the gut barrier and increased gut permeability directly or indirectly. Then inflammatory cytokines, immunologic factors, as well as microbial metabolites such as bile acids (BA) and short-chain fatty acids (SCFAs), might enter the enterohepatic circulation *via* a weak gut barrier to finally induce systematic lesion.

Existing findings demonstrate that Pb in gastrointestinal tracts have both direct and indirect effects on the gut. On the one hand, the intestinal epithelium and tight junctions are the targets for Pb toxicity, of which the damage and disruption together with Pb-induced inflammation and immune dysregulation in the local intestine may lead to increased gut permeability to macromolecules. On the other hand, Pb can also cause dysbiosis of intestinal microbiome, which are usually the first “victims” in gut, thereby compromising the function of gut barrier or change the expression of multiple microbial metabolites. Furthermore, Pb-induced alteration in intestinal microbiome can lead to gut permeability impairment, while disrupted gut barrier may in turn have impacts on microbial diversity, construction, and metabolites. Based on the subsequent alterations of both gut permeability and intestinal microbiome induced by Pb, we hypothesis that Pb exposure may cause an increased production of LPS which may be derived from dysbiosis of gut microbiota and damaged gut wall, thereby resulting in endotoxemia, through the impaired gut barrier. In addition, impairment of gut barrier may lead to bacterial translocation into other tissues, which may further induce endotoxemia and infections in the host. Finally, all the turbulent microbial metabolites, inflammatory cytokines, and immunologic factors enter the blood system, then go through the enterohepatic circulation, which may exert their functions on multiple target organs (Figure 1). Numerous studies have documented that LPS plays a vital role in the development of various diseases such as metabolic diseases (Saito et al., 2007), cardiovascular diseases (Neves et al., 2013; Troseid, 2017), liver, and kidney damage (McIntyre et al., 2011; Perea et al., 2017). Disturbed microbial metabolites are also involved in diseases by interacting with host metabolism. Considerable literature have documented quite a few probiotics as effective detoxicant, and a feasible treatment strategy for Pb toxicity. However, most of the studies focused on the general alteration of microbial communities by Pb, not the specific strain. In view of this, the metagenome and metabonome may need additional examinations to explore some key microbes and their functions on gut and other organs. The related mechanisms of Pb toxicity on gut and the further damage to other organs are not fully elucidated (especially Pb-induced alteration of LPS and related microbial metabolites). Moreover, further investigations on efficient mechanisms of probiotics against Pb are needed.

## Author Contributions

WL wrote the manuscript. HF, IM, and XW revised the manuscript. SZ, SX, and CZ discussed the manuscript. FY designed, revised, and finalized the manuscript. All the authors contributed to the article and approved the submitted version.

## Conflict of Interest

The authors declare that the research was conducted in the absence of any commercial or financial relationships that could be construed as a potential conflict of interest.

## Supplementary Material

Supplementary Figure 1 Pb toxicity on gut barrier. Pb can damage the gut barrier by inducing oxidative stress and decreasing tight junctions such as ZO-1, claudin-1, and occludin.

Supplementary Figure 2 Probiotics prevent Pb toxicity and improve gut health. Probiotics prevent Pb’s toxicity by uptaking and binding with it directly, or by producing SCFAs, increasing bile flow, upregulating the expression of tight junction, and reversing adverse reactions.

Supplementary Figure 3 Pb toxicity on ion transportation. At the time of lead exposure, lead enters inside the cell through Ca^2+^ channel and binds with calmodulin in place of Ca^2+^, thereby altering the cellular function.

Supplementary Figure 4 α diversity of gut microbiome affected by Pb.

## References

[B1] Adak A. Khan M. R. (2019). An insight into gut microbiota and its functionalities. *Cell. Mol. Sci.* 76 473–493. 10.1007/s00018-018-2943-4 30317530 PMC11105460

[B2] Ahamed M. Siddiqui M. K. (2007). Low level lead exposure and oxidative stress: current opinions. *Clin. Chim. Acta* 383 57–64. 10.1016/j.cca.2007.04.024 17573057

[B3] Al Osman M. Yang F. Massey I. Y. (2019). Exposure routes and health effects of heavy metals on children. *Biometals* 32 563–573. 10.1007/s10534-019-00193-5 30941546

[B4] Aldridge K. E. (1995). The occurrence, virulence, and antimicrobial resistance of anaerobes in polymicrobial infections. *Am. J. Surg.* 169(5A Suppl) 2S–7S. 7755163

[B5] Bae S. Ulrich C. M. Neuhouser M. L. Malysheva O. Bailey L. B. Xiao L. (2014). Plasma choline metabolites and colorectal cancer risk in the Women’s health initiative observational study. *Cancer Res.* 74 7442–7452. 10.1158/0008-5472.can-14-1835 25336191 PMC4268282

[B6] Baker F. Papiska H. Campbell L. L. (1962). Choline fermentation by Desulfovibrio desulfuricans. *J. Bacteriol.* 84 973–978. 10.1128/jb.84.5.973-978.1962 13969140 PMC277997

[B7] Becattini S. Taur Y. Pamer E. G. (2016). Antibiotic-induced changes in the intestinal microbiota and disease. *Trends Mol. Med.* 22 458–478. 10.1016/j.molmed.2016.04.003 27178527 PMC4885777

[B8] Bellinger D. C. (2016). Lead contamination in flint–an abject failure to protect public health. *N. Engl. J. Med.* 374 1101–1103. 10.1056/nejmp1601013 26863114

[B9] Beyenal H. Lewandowski Z. (2004). Dynamics of lead immobilization in sulfate reducing biofilms. *Water Res.* 38 2726–2736. 10.1016/j.watres.2004.03.023 15207603

[B10] Bhakta J. N. Munekage Y. Ohnishi K. Jana B. B. (2012). Isolation and identification of cadmium- and lead-resistant lactic acid bacteria for application as metal removing probiotic. *Int. J. Environ. Sci. Technol.* 9 433–440. 10.1007/s13762-012-0049-3

[B11] Bisanz J. E. Enos M. K. Mwanga J. R. Changalucha J. Burton J. P. Gloor G. B. (2014). Randomized open-label pilot study of the influence of probiotics and the gut microbiome on toxic metal levels in Tanzanian pregnant women and school children. *mBio* 5:e01580-14. 10.1128/mBio.01580-14 PMC4196227 25293764

[B12] Breton J. Daniel C. Vignal C. Body-Malapel M. Garat A. Ple C. (2016). Does oral exposure to cadmium and lead mediate susceptibility to colitis? The dark-and-bright sides of heavy metals in gut ecology. *Sci. Rep.* 6:19200. 10.1038/srep19200 PMC4707487 26752005

[B13] Breton J. Le Clere K. Daniel C. Sauty M. Nakab L. Chassat T. (2013a). Chronic ingestion of cadmium and lead alters the bioavailability of essential and heavy metals, gene expression pathways and genotoxicity in mouse intestine. *Arch. Toxicol.* 87 1787–1795. 10.1007/s00204-013-1032-6 23503628

[B14] Breton J. Massart S. Vandamme P. De Brandt E. Pot B. Foligne B. (2013b). Ecotoxicology inside the gut: impact of heavy metals on the mouse microbiome. *BMC Pharmacol. Toxicol.* 14:62. 10.1186/2050-6511-14-62 PMC3874687 24325943

[B15] Burger-van Paassen N. Vincent A. Puiman P. J. van der Sluis M. Bouma J. Boehm G. (2009). The regulation of intestinal mucin MUC2 expression by short-chain fatty acids: implications for epithelial protection. *Biochem. J.* 420 211–219. 10.1042/bj20082222 19228118

[B16] Cavalcante-Silva L. H. Galvao J. G. da Silva J. S. de Sales-Neto J. M. Rodrigues-Mascarenhas S. (2015). Obesity-driven gut microbiota inflammatory pathways to metabolic syndrome. *Front. Physiol.* 6:341. 10.3389/fphys.2015.00341 PMC4652019 26635627

[B17] Cheng D. Li H. Zhou J. Wang S. (2019). Chlorogenic acid relieves lead-induced cognitive impairments and hepato-renal damage via regulating the dysbiosis of the gut microbiota in mice. *Food Funct.* 10 681–690. 10.1039/c8fo01755g 30657151

[B18] Cho C. H. (2001). Current roles of nitric oxide in gastrointestinal disorders. *J. Physiol. Paris* 95 253–256. 10.1016/s0928-4257(01)00034-1 11595446

[B19] Clark H. F. Hausladen D. M. Brabander D. J. (2008). Urban gardens: lead exposure, recontamination mechanisms, and implications for remediation design. *Environ. Res.* 107 312–319. 10.1016/j.envres.2008.03.003 18456252

[B20] Clemente J. C. Ursell L. K. Parfrey L. W. Knight R. (2012). The impact of the gut microbiota on human health: an integrative view. *Cell* 148 1258–1270. 10.1016/j.cell.2012.01.035 22424233 PMC5050011

[B21] Cryan J. F. Dinan T. G. (2012). Mind-altering microorganisms: the impact of the gut microbiota on brain and behaviour. *Nat. Rev. Neurosci.* 13 701–712. 10.1038/nrn3346 22968153

[B22] Dabke K. Hendrick G. Devkota S. (2019). The gut microbiome and metabolic syndrome. *J. Clin. Invest.* 129 4050–4057. 10.1172/jci129194 31573550 PMC6763239

[B23] Daisley B. A. Monachese M. Trinder M. Bisanz J. E. Chmiel J. A. Burton J. P. (2019). Immobilization of cadmium and lead by *Lactobacillus rhamnosus* GR-1 mitigates apical-to-basolateral heavy metal translocation in a Caco-2 model of the intestinal epithelium. *Gut Microbes* 10 321–333. 10.1080/19490976.2018.1526581 30426826 PMC6546314

[B24] Datko-Williams L. Wilkie A. Richmond-Bryant J. (2014). Analysis of U.S. soil lead (Pb) studies from 1970 to 2012. *Sci. Total Environ.* 468-469 854–863. 10.1016/j.scitotenv.2013.08.089 24076506

[B25] Eggers S. Safdar N. Sethi A. K. Suen G. Peppard P. E. Kates A. E. (2019). Urinary lead concentration and composition of the adult gut microbiota in a cross-sectional population-based sample. *Environ. Int.* 133:105122. 10.1016/j.envint.2019.105122 31518933 PMC7230144

[B26] Evens A. Hryhorczuk D. Lanphear B. P. Rankin K. M. Lewis D. A. Forst L. (2015). The impact of low-level lead toxicity on school performance among children in the Chicago Public Schools: a population-based retrospective cohort study. *Environ. Health* 14:21. 10.1186/s12940-015-0008-9 PMC4387706 25889033

[B27] Fackelmann G. Sommer S. (2019). Microplastics and the gut microbiome: how chronically exposed species may suffer from gut dysbiosis. *Mar. Pollut. Bull.* 143 193–203. 10.1016/j.marpolbul.2019.04.030 31789155

[B28] Feng J. Cavallero S. Hsiai T. Li R. (2020). Impact of air pollution on intestinal redox lipidome and microbiome. *Free Radic. Biol. Med.* 151 99–110. 10.1016/j.freeradbiomed.2019.12.044 31904545

[B29] Flora G. Gupta D. Tiwari A. (2012). Toxicity of lead: a review with recent updates. *Interdiscip. Toxicol.* 5 47–58. 10.2478/v10102-012-0009-2 23118587 PMC3485653

[B30] Gao B. Chi L. Mahbub R. Bian X. Tu P. Ru H. (2017). Multi-omics reveals that lead exposure disturbs gut microbiome development, key metabolites, and metabolic pathways. *Chem. Res. Toxicol.* 30 996–1005. 28234468 10.1021/acs.chemrestox.6b00401 PMC5654721

[B31] Gao D. Mondal T. K. Lawrence D. A. (2007). Lead effects on development and function of bone marrow-derived dendritic cells promote Th2 immune responses. *Toxicol. Appl. Pharmacol.* 222 69–79. 10.1016/j.taap.2007.04.001 17512567 PMC2744586

[B32] Gao R. Gao Z. Huang L. Qin H. (2017). Gut microbiota and colorectal cancer. *Eur. J. Clin. Microbiol. Infect. Dis.* 36 757–769. 28063002 10.1007/s10096-016-2881-8 PMC5395603

[B33] Gilbert S. G. Weiss B. (2006). A rationale for lowering the blood lead action level from 10 to 2 microg/dL. *Neurotoxicology* 27 693–701. 10.1016/j.neuro.2006.06.008 16889836 PMC2212280

[B34] Giri S. S. Jun J. W. Yun S. Kim H. J. Kim S. G. Kang J. W. (2019). Characterisation of lactic acid bacteria isolated from the gut of *Cyprinus carpio* that may be effective against lead toxicity. *Probiotics Antimicrob. Proteins* 11 65–73. 10.1007/s12602-017-9367-6 29285742

[B35] Giri S. S. Yun S. Jun J. W. Kim H. J. Kim S. G. Kang J. W. (2018). Therapeutic effect of intestinal autochthonous *Lactobacillus reuteri* P16 against waterborne lead toxicity in *Cyprinus carpio*. *Front. Immunol.* 9:1824. 10.3389/fimmu.2018.01824 PMC6090060 30131809

[B36] Halttunen T. Salminen S. Tahvonen R. (2007). Rapid removal of lead and cadmium from water by specific lactic acid bacteria. *Int. J. Food Microbiol.* 114 30–35. 10.1016/j.ijfoodmicro.2006.10.040 17184867

[B37] He X. Wu J. Yuan L. Lin F. Yi J. Li J. (2017). Lead induces apoptosis in mouse TM3 Leydig cells through the Fas/FasL death receptor pathway. *Environ. Toxicol. Pharmacol.* 56 99–105. 10.1016/j.etap.2017.08.034 28889079

[B38] Hofmann A. F. Hagey L. R. (2014). Key discoveries in bile acid chemistry and biology and their clinical applications: history of the last eight decades. *J. Lipid Res.* 55 1553–1595. 24838141 10.1194/jlr.R049437 PMC4109754

[B39] Holmes E. Li J. V. Athanasiou T. Ashrafian H. Nicholson J. K. (2011). Understanding the role of gut microbiome-host metabolic signal disruption in health and disease. *Trends Microbiol.* 19 349–359. 10.1016/j.tim.2011.05.006 21684749

[B40] Huang H. Wang Y. An Y. Jiao W. Xu Y. Han Q. (2019). Selenium alleviates oxidative stress and autophagy in lead-treated chicken testes. *Theriogenology* 131 146–152. 10.1016/j.theriogenology.2019.03.015 30965207

[B41] Jin Y. Wu S. Zeng Z. Fu Z. (2017). Effects of environmental pollutants on gut microbiota. *Environ. Pollut.* 222 1–9. 10.1007/978-981-15-4759-1_1 28086130

[B42] Jomova K. Valko M. (2011). Advances in metal-induced oxidative stress and human disease. *Toxicology* 283 65–87. 10.1016/j.tox.2011.03.001 21414382

[B43] Jones B. V. Begley M. Hill C. Gahan C. G. Marchesi J. R. (2008). Functional and comparative metagenomic analysis of bile salt hydrolase activity in the human gut microbiome. *Proc. Natl. Acad. Sci. U.S.A.* 105 13580–13585. 10.1073/pnas.0804437105 18757757 PMC2533232

[B44] Juturu V. Wu J. C. (2018). Microbial production of bacteriocins: latest research development and applications. *Biotechnol. Adv.* 36 2187–2200. 10.1016/j.biotechadv.2018.10.007 30385277

[B45] Koh A. De Vadder F. Kovatcheva-Datchary P. Bäckhed F. J. C. (2016). From dietary fiber to host physiology: short-chain fatty acids as key bacterial metabolites. *Cell* 165 1332–1345. 10.1016/j.cell.2016.05.041 27259147

[B46] Kou H. Fu Y. He Y. Jiang J. Gao X. Zhao H. (2019). Chronic lead exposure induces histopathological damage, microbiota dysbiosis and immune disorder in the cecum of female Japanese quails (*Coturnix japonica*). *Ecotoxicol. Environ. Saf.* 183:109588. 10.1016/j.ecoenv.2019.109588 31450035

[B47] Lamattina L. Garcia-Mata C. Graziano M. Pagnussat G. (2003). Nitric oxide: the versatility of an extensive signal molecule. *Annu. Rev. Plant Biol.* 54 109–136. 10.1146/annurev.arplant.54.031902.134752 14502987

[B48] Lanphear B. P. Rauch S. Auinger P. Allen R. W. Hornung R. W. (2018). Low-level lead exposure and mortality in US adults: a population-based cohort study. *Lancet Public Health* 3 e177–e184. 29544878 10.1016/S2468-2667(18)30025-2

[B49] Ley R. E. Peterson D. A. Gordon J. I. (2006). Ecological and evolutionary forces shaping microbial diversity in the human intestine. *Cell* 124 837–848. 10.1016/j.cell.2006.02.017 16497592

[B50] Li D. Xu X. Yu H. Han X. (2017). Characterization of Pb2+ biosorption by psychrotrophic strain *Pseudomonas* sp. I3 isolated from permafrost soil of Mohe wetland in Northeast China. *J. Environ. Manage.* 196 8–15. 10.1016/j.jenvman.2017.02.076 28284141

[B51] Li T. Zhang S. Tan Z. Dai Y. (2017). Trend of childhood blood lead levels in cities of China in recent 10 years. *Environ. Sci. Pollut. Res. Int.* 24 5824–5830. 10.1007/s11356-016-8335-0 28054269

[B52] Liew W. P. Mohd-Redzwan S. (2018). Mycotoxin: its impact on gut health and microbiota. *Front. Cell Infect. Microbiol.* 8:60. 10.3389/fcimb.2018.00060 PMC5834427 29535978

[B53] Liu D. J. Wu J. Liu Y. Q. Ouyang L. Wang J. Y. (2014). [Effects of lead exposure on 18 elements in blood and excretions in rats]. *Beijing Da Xue Xue Bao Yi Xue Ban* 46 232–236. 24743812

[B54] Lo Presti A. Zorzi F. Del Chierico F. Altomare A. Cocca S. Avola A. (2019). Fecal and mucosal microbiota profiling in irritable bowel syndrome and inflammatory bowel disease. *Front. Microbiol.* 10:1655. 10.3389/fmicb.2019.01655 PMC6650632 31379797

[B55] Ma C. Han M. Heinrich B. Fu Q. Zhang Q. Sandhu M. (2018). Gut microbiome-mediated bile acid metabolism regulates liver cancer via NKT cells. *Science* 360:eaan5931. 10.1126/science.aan5931 29798856 PMC6407885

[B56] Mangiola F. Ianiro G. Franceschi F. Fagiuoli S. Gasbarrini G. Gasbarrini A. (2016). Gut microbiota in autism and mood disorders. *World J. Gastroenterol.* 22 361–368. 10.3748/wjg.v22.i1.361 26755882 PMC4698498

[B57] Marchesi J. R. Adams D. H. Fava F. Hermes G. D. Hirschfield G. M. Hold G. (2016). The gut microbiota and host health: a new clinical frontier. *Gut* 65 330–339. 10.1136/gutjnl-2015-309990 26338727 PMC4752653

[B58] McIntyre C. W. Harrison L. E. Eldehni M. T. Jefferies H. J. Szeto C. C. John S. G. (2011). Circulating endotoxemia: a novel factor in systemic inflammation and cardiovascular disease in chronic kidney disease. *Clin. J. Am. Soc. Nephrol.* 6 133–141. 10.2215/cjn.04610510 20876680 PMC3022234

[B59] McLellan S. L. Newton R. J. Vandewalle J. L. Shanks O. C. Huse S. M. Eren A. M. (2013). Sewage reflects the distribution of human faecal *L achnospiraceae*. *Environ. Microbiol.* 15 2213–2227. 10.1111/1462-2920.12092 23438335 PMC4043349

[B60] Metryka E. Chibowska K. Gutowska I. Falkowska A. Kupnicka P. Barczak K. (2018). Lead (Pb) exposure enhances expression of factors associated with inflammation. *Int. J. Mol. Sci.* 19:1813. 10.3390/ijms19061813 29925772 PMC6032409

[B61] Mielke H. W. Anderson J. C. Berry K. J. Mielke P. W. Chaney R. L. Leech M. (1983). Lead concentrations in inner-city soils as a factor in the child lead problem. *Am. J. Public Health* 73 1366–1369. 10.2105/ajph.73.12.1366 6638229 PMC1651267

[B62] Mielke H. W. Blake B. Burroughs S. Hassinger N. (1984). Urban lead levels in Minneapolis: the case of the Hmong children. *Environ. Res.* 34 64–76. 10.1016/0013-9351(84)90076-8 6723610

[B63] Mitchell R. G. Spliethoff H. M. Ribaudo L. N. Lopp D. M. Shayler H. A. Marquez-Bravo L. G. (2014). Lead (Pb) and other metals in New York City community garden soils: factors influencing contaminant distributions. *Environ. Pollut.* 187 162–169. 10.1016/j.envpol.2014.01.007 24502997 PMC3983949

[B64] Monast C. S. Telesco S. Li K. Hayden K. Brodmerkel C. J. G. (2016). Su1217 the role of the microbiome in clinical response to golimumab in ulcerative colitis. *Gastroenterology* 150:S498.

[B65] Mostafa G. A. El-Shahawi H. H. Mokhtar A. (2009). Blood lead levels in Egyptian children from high and low lead-polluted areas: impact on cognitive function. *Acta Neurol. Scand.* 120 30–37. 10.1111/j.1600-0404.2009.01155.x 19222397

[B66] Navas-Acien A. Guallar E. Silbergeld E. K. Rothenberg S. J. (2007). Lead exposure and cardiovascular disease–a systematic review. *Environ. Health Perspect.* 115 472–482. 10.1289/ehp.9785 17431501 PMC1849948

[B67] Neves A. L. Coelho J. Couto L. Leite-Moreira A. Roncon-Albuquerque R. Jr. (2013). Metabolic endotoxemia: a molecular link between obesity and cardiovascular risk. *J. Mol. Endocrinol.* 51 R51–R64. 23943858 10.1530/JME-13-0079

[B68] Ni J. Wu G. D. Albenberg L. Tomov V. T. (2017). Gut microbiota and IBD: causation or correlation? *Nat. Rev. Gastroenterol. Hepatol.* 14 573–584. 10.1038/nrgastro.2017.88 28743984 PMC5880536

[B69] Nishida A. Inoue R. Inatomi O. Bamba S. Naito Y. Andoh A. (2018). Gut microbiota in the pathogenesis of inflammatory bowel disease. *Clin. J. Gastroenterol.* 11 1–10. 29285689 10.1007/s12328-017-0813-5

[B70] Org E. Mehrabian M. Lusis A. J. (2015). Unraveling the environmental and genetic interactions in atherosclerosis: central role of the gut microbiota. *Atherosclerosis* 241 387–399. 10.1016/j.atherosclerosis.2015.05.035 26071662 PMC4510029

[B71] O’Toole P. W. Jeffery I. B. (2015). Gut microbiota and aging. *Science* 350 1214–1215. 26785481 10.1126/science.aac8469

[B72] Park S. J. Kim J. Lee J. S. Rhee S. K. Kim H. (2014). Characterization of the fecal microbiome in different swine groups by high-throughput sequencing. *Anaerobe* 28 157–162. 10.1016/j.anaerobe.2014.06.002 24954845

[B73] Patsiou D. Del Rio-Cubilledo C. Catarino A. I. Summers S. Mohd Fahmi A. Boyle D. (2020). Exposure to Pb-halide perovskite nanoparticles can deliver bioavailable Pb but does not alter endogenous gut microbiota in zebrafish. *Sci. Total Environ.* 715:136941. 10.1016/j.scitotenv.2020.136941 32041050

[B74] Perea L. Coll M. Sanjurjo L. Blaya D. Taghdouini A. E. Rodrigo-Torres D. (2017). Pentraxin-3 modulates lipopolysaccharide-induced inflammatory response and attenuates liver injury. *Hepatology* 66 953–968. 10.1002/hep.29215 28422322 PMC5570620

[B75] Raghuvanshi R. Chaudhari A. Kumar G. N. (2017). 2-Ketogluconic acid and pyrroloquinoline quinone secreting probiotic *Escherichia coli* Nissle 1917 as a dietary strategy against heavy metal induced damage in rats. *J. Funct. Foods* 37 541–552. 10.1016/j.jff.2017.08.013

[B76] Rashid A. Bhat R. A. Qadri H. Mehmood M. A. Shafiq Ur R. (2019). Environmental and socioeconomic factors induced blood lead in children: an investigation from Kashmir, India. *Environ. Monit. Assess.* 191:76. 10.1007/s10661-019-7220-y 30648205

[B77] Riaz M. Mahmood Z. Shahid M. Saeed M. U. I Tahir M. Shah S. A. (2016). Impact of reactive oxygen species on antioxidant capacity of male reproductive system. *Int. J. Immunopathol. Pharmacol.* 29 421–425. 10.1177/0394632015608994 26684624 PMC5806750

[B78] Ridlon J. M. Bajaj J. S. (2015). The human gut sterolbiome: bile acid-microbiome endocrine aspects and therapeutics. *Acta Pharm. Sin B* 5 99–105. 10.1016/j.apsb.2015.01.006 26579434 PMC4629220

[B79] Ridlon J. M. Kang D. J. Hylemon P. B. Bajaj J. S. (2015). Gut microbiota, cirrhosis, and alcohol regulate bile acid metabolism in the gut. *Dig. Dis.* 33 338–345. 10.1159/000371678 26045267 PMC4470395

[B80] Sadykov R. Digel I. Artmann A. T. Porst D. Linder P. Kayser P. (2009). Oral lead exposure induces dysbacteriosis in rats. *J. Occup. Health* 51 64–73. 10.1539/joh.m8009 19096199

[B81] Saito T. Hayashida H. Furugen R. (2007). Comment on: Cani et al. (2007) Metabolic endotoxemia initiates obesity and insulin resistance: diabetes 56:1761-1772. *Diabetes* 56:e20; author reply e21. 10.2337/db07-1181 18042755

[B82] Satcher D. S. (2000). The surgeon general on the continuing tragedy of childhood lead poisoning. *Public Health Rep.* 115 579–580. 10.1093/phr/115.6.579 11354340 PMC1308629

[B83] Schwab C. Berry D. Rauch I. Rennisch I. Ramesmayer J. Hainzl E. (2014). Longitudinal study of murine microbiota activity and interactions with the host during acute inflammation and recovery. *ISME J.* 8 1101–1114. 10.1038/ismej.2013.223 24401855 PMC3996699

[B84] Shao M. Zhu Y. (2020). Long-term metal exposure changes gut microbiota of residents surrounding a mining and smelting area. *Sci. Rep.* 10:4453. 10.1038/s41598-020-61143-7 PMC7064573 32157109

[B85] Sharon G. Garg N. Debelius J. Knight R. Dorrestein P. C. Mazmanian S. K. (2014). Specialized metabolites from the microbiome in health and disease. *Cell Metab.* 20 719–730. 10.1016/j.cmet.2014.10.016 25440054 PMC4337795

[B86] Stojanov S. Berlec A. Štrukelj B. J. M. (2020). The influence of probiotics on the Firmicutes/Bacteroidetes ratio in the treatment of obesity and inflammatory bowel disease. *Microorganisms* 8:1715. 10.3390/microorganisms8111715 PMC7692443 33139627

[B87] Szolnoki Z. Farsang A. Puskas I. (2013). Cumulative impacts of human activities on urban garden soils: origin and accumulation of metals. *Environ. Pollut.* 177 106–115. 10.1016/j.envpol.2013.02.007 23500047

[B88] Tamanai-Shacoori Z. Smida I. Bousarghin L. Loreal O. Meuric V. Fong S. B. (2017). Roseburia spp.: a marker of health? *Future Microbiol.* 12 157–170. 28139139 10.2217/fmb-2016-0130

[B89] Tang W. W. Wang Z. Levison B. S. Koeth R. A. Britt E. B. Fu X. (2013). Intestinal microbial metabolism of phosphatidylcholine and cardiovascular risk. *N. Engl. J. Med.* 368 1575–1584. 10.1056/nejmoa1109400 23614584 PMC3701945

[B90] Tian F. Zhai Q. Zhao J. Liu X. Wang G. Zhang H. (2012). Lactobacillus plantarum CCFM8661 alleviates lead toxicity in mice. *Biol. Trace Elem. Res.* 150 264–271. 10.1007/s12011-012-9462-1 22684513

[B91] Tomczok J. Grzybek H. Sliwa W. Panz B. (1988). Ultrastructural aspects of the small intestinal lead toxicology. Part I: surface ultrastructure of the small intestine mucosa in rats with lead acetate poisoning. *Exp. Pathol.* 35 49–55. 10.1016/s0232-1513(88)80122-1 3229465

[B92] Topcu A. Bulat T. (2010). Removal of cadmium and lead from aqueous solution by *Enterococcus faecium* strains. *J. Food Sci.* 75 T13–T17. 20492209 10.1111/j.1750-3841.2009.01429.x

[B93] Tremaroli V. Backhed F. (2012). Functional interactions between the gut microbiota and host metabolism. *Nature* 489 242–249. 10.1038/nature11552 22972297

[B94] Tripathi A. Debelius J. Brenner D. A. Karin M. Loomba R. Schnabl B. (2018). Publisher Correction: the gut-liver axis and the intersection with the microbiome. *Nat. Rev. Gastroenterol. Hepatol.* 15:785. 10.1038/s41575-018-0031-8 29785003 PMC7133393

[B95] Troseid M. (2017). Gut microbiota and acute coronary syndromes: ready for use in the emergency room? *Eur. Heart J.* 38 825–827. 10.1093/eurheartj/ehx005 28159962 PMC5381592

[B96] Tsoi M. F. Cheung C. L. Cheung T. T. Cheung B. M. (2016). Continual decrease in blood lead level in Americans: United States national health nutrition and examination survey 1999-2014. *Am. J. Med.* 129 1213–1218. 10.1016/j.amjmed.2016.05.042 27341956

[B97] Turnbaugh P. J. Hamady M. Yatsunenko T. Cantarel B. L. Duncan A. Ley R. E. (2009). A core gut microbiome in obese and lean twins. *Nature* 457 480–484. 10.1038/nature07540 19043404 PMC2677729

[B98] Velmurugan G. Ramprasath T. Gilles M. Swaminathan K. Ramasamy S. (2017). Gut microbiota, endocrine-disrupting chemicals, and the diabetes epidemic. *Trends Endocrinol. Metab.* 28 612–625. 10.1016/j.tem.2017.05.001 28571659

[B99] Ver Heul A. Planer J. Kau A. L. (2019). The human microbiota and asthma. *Clin. Rev. Allergy Immunol.* 57 350–363. 10.1007/s12016-018-8719-7 30426401 PMC7449604

[B100] Wahlström A. Sayin S. I. Marschall H.-U. Bäckhed F. (2016). Intestinal crosstalk between bile acids and microbiota and its impact on host metabolism. *Cell Metab.* 24 41–50. 10.1016/j.cmet.2016.05.005 27320064

[B101] Wang L. Tang L. Feng Y. Zhao S. Han M. Zhang C. (2020). A purified membrane protein from *Akkermansia muciniphila* or the pasteurised bacterium blunts colitis associated tumourigenesis by modulation of CD8+ T cells in mice. *Gut.* 69 1988–1997. 32169907 10.1136/gutjnl-2019-320105 PMC7569398

[B102] Wang R. X. Lee J. S. Campbell E. L. Colgan S. P. (2020). Microbiota-derived butyrate dynamically regulates intestinal homeostasis through regulation of actin-associated protein synaptopodin. *Proc. Natl. Acad. Sci. U.S.A.* 117 11648–11657. 10.1073/pnas.1917597117 32398370 PMC7260972

[B103] Watson W. A. Litovitz T. L. Rodgers G. C. Jr. Klein-Schwartz W. Reid N. Youniss J. (2005). 2004 annual report of the American association of poison control centers toxic exposure surveillance system. *Am. J. Emerg. Med.* 23 589–666. 10.1016/j.ajem.2005.05.001 16140178

[B104] Wong J. M. de Souza R. Kendall C. W. Emam A. Jenkins D. J. (2006). Colonic health: fermentation and short chain fatty acids. *J. Clin. Gastroenterol.* 40 235–243. 16633129 10.1097/00004836-200603000-00015

[B105] Wu H. Esteve E. Tremaroli V. Khan M. T. Caesar R. Manneras-Holm L. (2017). Metformin alters the gut microbiome of individuals with treatment-naive type 2 diabetes, contributing to the therapeutic effects of the drug. *Nat. Med.* 23 850–858. 10.1038/nm.4345 28530702

[B106] Wu J. Wen X. W. Faulk C. Boehnke K. Zhang H. Dolinoy D. C. (2016). Perinatal lead exposure alters gut microbiota composition and results in sex-specific bodyweight increases in adult mice. *Toxicol. Sci.* 151 324–333. 10.1093/toxsci/kfw046 26962054 PMC4880136

[B107] Xia J. Jin C. Pan Z. Sun L. Fu Z. Jin Y. (2018a). Chronic exposure to low concentrations of lead induces metabolic disorder and dysbiosis of the gut microbiota in mice. *Sci. Total Environ.* 63 439–448. 10.1016/j.scitotenv.2018.03.053 29529432

[B108] Xia J. Lu L. Jin C. Wang S. Zhou J. Ni Y. (2018b). Effects of short term lead exposure on gut microbiota and hepatic metabolism in adult zebrafish. *Comp. Biochem. Physiol. C Toxicol. Pharmacol.* 209 1–8. 10.1016/j.cbpc.2018.03.007 29574035

[B109] Yi Y. J. Lim J. M. Gu S. Lee W. K. Oh E. Lee S. M. (2017). Potential use of lactic acid bacteria *Leuconostoc mesenteroides* as a probiotic for the removal of Pb(II) toxicity. *J. Microbiol.* 55 296–303. 10.1007/s12275-017-6642-x 28361342

[B110] Yu L. X. Schwabe R. F. (2017). The gut microbiome and liver cancer: mechanisms and clinical translation. *Nat. Rev. Gastroenterol. Hepatol.* 14 527–539. 10.1038/nrgastro.2017.72 28676707 PMC6467288

[B111] Yuan X. Pan Z. Jin C. Ni Y. Fu Z. Jin Y. (2019). Gut microbiota: an underestimated and unintended recipient for pesticide-induced toxicity. *Chemosphere* 227 425–434. 10.1016/j.chemosphere.2019.04.088 31003127

[B112] Zhai Q. Li T. Yu L. Xiao Y. Feng S. Wu J. (2017a). Effects of subchronic oral toxic metal exposure on the intestinal microbiota of mice. *Sci. Bull.* 62 831–840. 10.1016/j.scib.2017.01.031 36659316

[B113] Zhai Q. Liu Y. Wang C. Qu D. Zhao J. Zhang H. (2019a). *Lactobacillus plantarum* CCFM8661 modulates bile acid enterohepatic circulation and increases lead excretion in mice. *Food Funct.* 10 1455–1464. 10.1039/c8fo02554a 30768114

[B114] Zhai Q. Qu D. Feng S. Yu Y. Yu L. Tian F. (2019b). Oral supplementation of lead-intolerant intestinal microbes protects against lead (Pb) toxicity in mice. *Front. Microbiol.* 10:3161. 10.3389/fmicb.2019.03161 PMC6987320 32038590

[B115] Zhai Q. Wang H. Tian F. Zhao J. Zhang H. Chen W. (2017b). Dietary *Lactobacillus plantarum* supplementation decreases tissue lead accumulation and alleviates lead toxicity in Nile tilapia (*Oreochromis niloticus*). *Aquacult. Res.* 48 5094–5103. 10.1111/are.13326

[B116] Zhai Q. Wang J. Cen S. Zhao J. Zhang H. Tian F. (2019c). Modulation of the gut microbiota by a galactooligosaccharide protects against heavy metal lead accumulation in mice. *Food Funct.* 10 3768–3781. 10.1039/c9fo00587k 31180403

[B117] Zhai Q. Yang L. Zhao J. Zhang H. Tian F. Chen W. (2018). Protective effects of dietary supplements containing probiotics, micronutrients, and plant extracts against lead toxicity in mice. *Front. Microbiol.* 9:2134. 10.3389/fmicb.2018.02134 PMC6141689 30254621

[B118] Zhang Z. Zhang X.-X. Wu B. Yin J. Yu Y. Yang L. (2016). Comprehensive insights into microcystin-LR effects on hepatic lipid metabolism using cross-omics technologies. *J. Hazardous Mater.* 315 126–134. 10.1016/j.jhazmat.2016.05.011 27208774

[B119] Zmora N. Zeevi D. Korem T. Segal E. Elinav E. (2016). Taking it personally: personalized utilization of the human microbiome in health and disease. *Cell Host Microbe* 19 12–20. 10.1016/j.chom.2015.12.016 26764593

