# Supplementary figures and images for: Pb Toxicity on Gut Physiology and Microbiota
Source: Front Physiol. 2021 Mar 4;12:574913. doi: 10.3389/fphys.2021.574913 (PMC7970193; doi:10.3389/fphys.2021.574913)

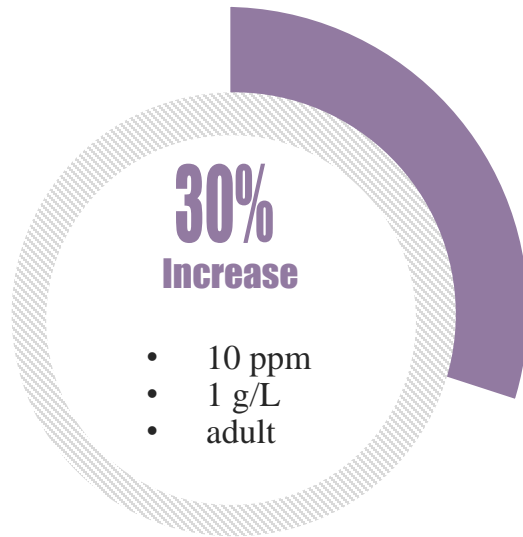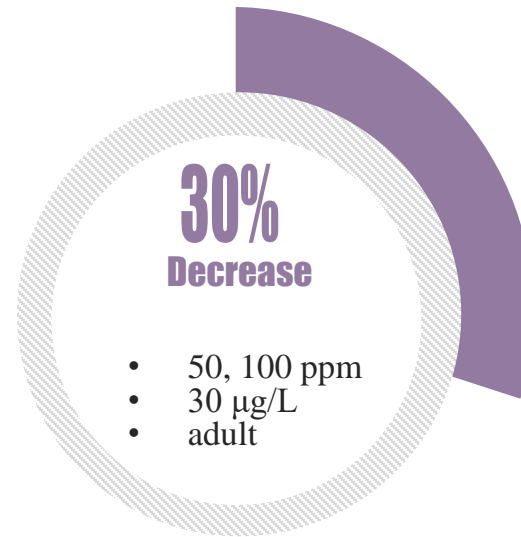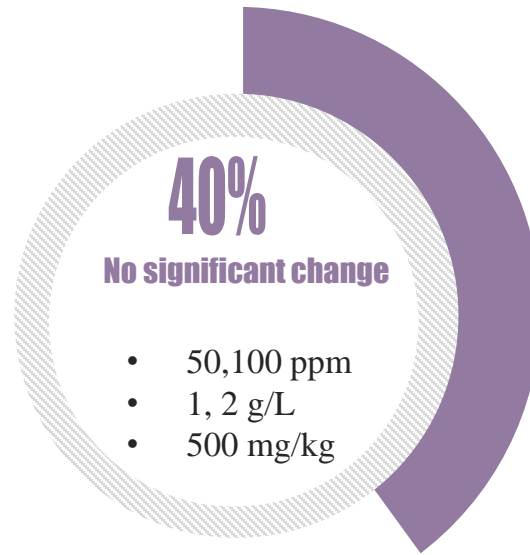

Supplement: Supplementary Figure 1 — Pb toxicity on gut barrier. Pb can damage the gut barrier by inducing oxidative stress and decreasing tight junctions such as ZO-1, claudin-1, and occludin. [file Data_Sheet_1.PDF]

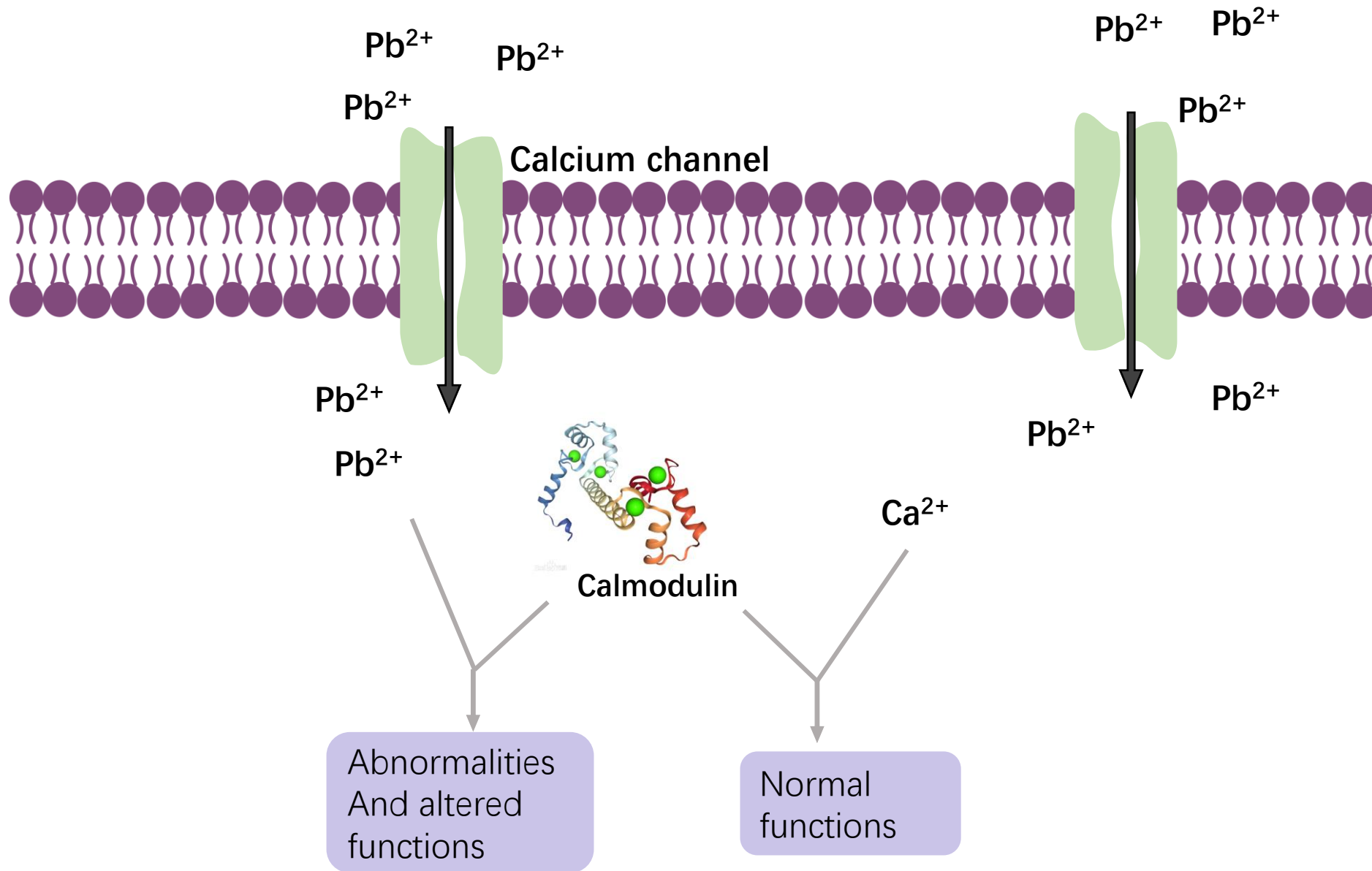

Supplement: Supplementary Figure 2 — Probiotics prevent Pb toxicity and improve gut health. Probiotics prevent Pb’s toxicity by uptaking and binding with it directly, or by producing SCFAs, increasing bile flow, upregulating the expression of tight junction, and reversing adverse reactions. [file Data_Sheet_2.PDF]

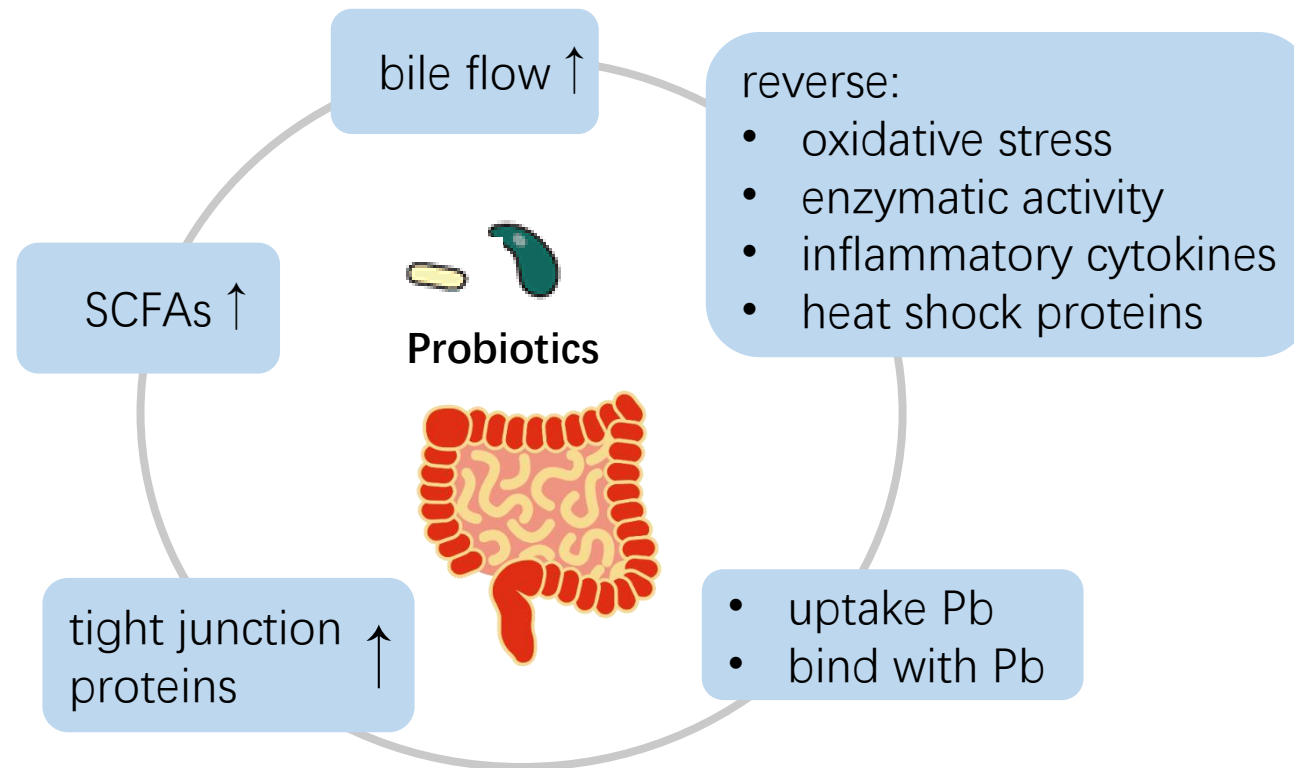

Supplement: Supplementary Figure 3 — Pb toxicity on ion transportation. At the time of lead exposure, lead enters inside the cell through Ca2+ channel and binds with calmodulin in place of Ca2+, thereby altering the cellular function. [file Data_Sheet_3.PDF]
