# Supplementary material for: Pb Toxicity on Gut Physiology and Microbiota
Source: Front Physiol. 2021 Mar 4;12:574913. doi: 10.3389/fphys.2021.574913 (PMC7970193; doi:10.3389/fphys.2021.574913)
Supplement: Supplementary Figure 4 — α diversity of gut microbiome affected by Pb. [file Data_Sheet_4.PDF]

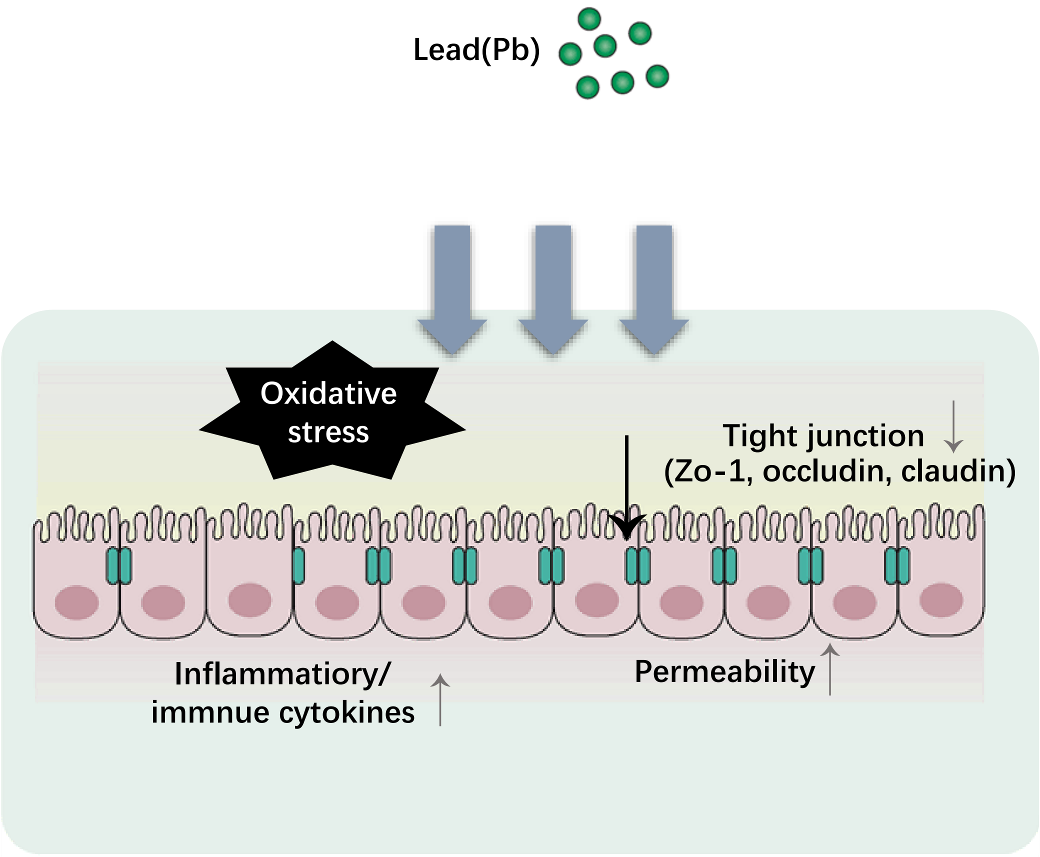
Lead(Pb)
Oxidative stress
Tight junction
(Zo-1, occludin, claudin)
Inflammatiory/
immnue cytokines
Permeability
